# Clinical and genetic characteristics predict outcomes of acute myeloid leukemia patients with FLT3 mutations receiving venetoclax‐based therapy

**DOI:** 10.1002/cam4.6885

**Published:** 2024-02-09

**Authors:** Guangyang Weng, Jingya Huang, Na An, Yu Zhang, Guopan Yu, Zhiqiang Sun, Dongjun Lin, Lan Deng, Xinquan Liang, Jie Xiao, Hongyu Zhang, Ziwen Guo, Xin He, Hua Jin, Qifa Liu, Xin Du

**Affiliations:** ^1^ Department of Hematology and Shenzhen Bone Marrow Transplantation Public Service Platform The First Affiliated Hospital of Shenzhen University, Shenzhen Second People's Hospital Shenzhen China; ^2^ Shenzhen Blood Center Shenzhen Guangdong China; ^3^ Department of Hematology and Shenzhen Bone Marrow Transplantation Public Service Platform, Shenzhen Institute of Hematology, Shenzhen Second People's Hospital The First Affiliated Hospital of Shenzhen University, Shenzhen University Health Sciences Center Shenzhen China; ^4^ Department of Hematology Nanfang Hospital, Southern Medical University Guangzhou China; ^5^ Department of Hematology, Shenzhen Hospital Southern Medical University Shenzhen China; ^6^ Department of Hematology the Seventh Affiliated Hospital of Sun Yat‐Sen University Shenzhen China; ^7^ Department of Hematology, Shanghai Ninth People's Hospital Shanghai Jiao Tong University School of Medicine Shanghai China; ^8^ Department of Hematology The First People's Hospital of Chenzhou Chenzhou China; ^9^ Department of Hematology Sun Yat‐Sen Memorial Hospital, Sun Yat‐Sen University Guangzhou China; ^10^ Department of Hematology Peking University Shenzhen Hospital Shenzhen China; ^11^ Department of Hematology Zhongshan City People's Hospital Zhongshan China

**Keywords:** acute myeloid leukemia, FLT3 mutations, genetic characteristics, hypomethylating agents, venetoclax

## Abstract

**Background:**

Acute myeloid leukemia (AML) is a heterogeneous disease, and its heterogeneity is associated with treatment response. Despite the demonstrated success of venetoclax (VEN)‐based therapy for AML, the effect of FLT3 mutations on the efficacy of the therapy is poorly understood. We aimed to compare the efficacy of VEN‐based therapy between FLT3‐mutated (FLT3^mut^) and FLT3 wild‐type (FLT3^wt^) patients and identify the predictors of efficacy in FLT3^mut^ patients.

**Methods:**

A total of 266 AML patients (127 newly diagnosed [ND] and 139 refractory/relapsed [R/R]) receiving VEN‐based regimens were enrolled in this study. A retrospective analysis was performed, and the treatment responses and overall survival (OS) of FLT3^mut^ and FLT3^wt^ patients were compared. Logistic regression and Cox proportional hazards model were applied to examine the clinical and genetic predictors of outcomes.

**Results:**

With a median of two cycles of VEN‐based therapy, for the ND AML cohort, the FLT3^mut^ group had a comparable composite complete remission (CRc) rate with the FLT3^wt^ group (79.3% vs. 61.2%, *p* = 0.072). For the R/R AML cohort, the FLT3^mut^ group exhibited a lower CRc rate than the FLT3^wt^ group. With a median follow‐up of 8.6 months (95% confidence interval [CI], 8.0–10), the median OS observed in the FLT3^mut^ and FLT3^wt^ groups for both cohorts were close (14.0 vs. 19.9 months, *p* = 0.356; 10.0 vs. 11.9 months, *p* = 0.680). For the ND AML cohort, in FLT3^mut^ patients, MRD‐positive and RNA‐splicing mutation predicted inferior survival (hazard ratio [HR], 10.3; 95% CI: 2.0–53.8; *p* = 0.006; HR 11.3; 95% CI: 1.2–109.3; *p* = 0.036, respectively). For the R/R AML cohort, in FLT3^mut^ patients, adverse ELN risk was associated with an inferior response (odds ratio [OR], 0.2; 95% CI: 0.1–0.8; *p* = 0.025), whereas NPM1 co‐mutation was associated with a superior response (57.1%; OR, 6.7; 95% CI: 1.5–30.1; *p* = 0.014). CR/CRi predicted a better survival (HR 0.2; 95% CI: 0.1–0.8; *p* = 0.029), while DNMT3A mutation predicted an inferior survival (HR, 4.6; 95% CI: 1.4–14.9; *p* = 0.011).

**Conclusions:**

FLT3 mutations may influence response to VEN‐based therapy in R/R AML patients but not in ND AML patients. Furthermore, clinical and genetic characteristics could predict outcomes of FLT3^mut^ patients receiving VEN‐based therapy.

## 1 INTRODUCTION

Venetoclax (VEN) has demonstrated successful application in newly diagnosed (ND) AML patients deemed ineligible for intensive chemotherapy due to its high response rate, longer overall survival, and low treatment‐related mortality.^1^, ^2^, ^3^ Currently, the combination of VEN with hypomethylating agents (HMA) or low‐dose cytarabine (LDAC) is recommended as a new standard of care for elderly or unfit AML patients.^4^ Encouragingly, VEN‐based regimens have also shown efficacy in patients with relapsed or refractory (R/R) AML.^5^, ^6^, ^7^, ^8^, ^9^ However, over 30% of ND AML patients and a majority of R/R AML patients still exhibit primary resistance to VEN‐based regimens, which may be attributed to the heterogeneity of AML, particularly in terms of genetic variations. Notably, mutations in the FMS‐like tyrosine kinase 3 (FLT3) gene observed in 25%–35% of AML patients have been definitively linked to leukemia progression, increased cell proliferation, and unfavorable prognosis.^10^, ^11^, ^12^ Therefore, exploring the impact of FLT3 mutations on the efficacy of VEN‐based regimens is of particular importance. Subgroup analyses conducted in Phase 3 clinical trials have provided insights into the influence of FLT3 mutations on treatment response.^2^, ^13^ However, there is no detailed comparison between FLT3‐mutated (FLT3^mut^) and FLT3 wild‐type (FLT3^wt^) patients in the extant literature. Thus, the impact of FLT3 mutations on the efficacy of VEN‐based therapy remains uncertain. Previous studies have reported varying composite complete remission (CRc) rates in FLT3^mut^ patients receiving VEN‐based regimens, ranging from 44% to 66.7%. A Phase 3 randomized study demonstrated a CR/CRi rate of 66.7% in 42 FLT3^mut^ patients,^2^, ^14^ and a prospective trial showed a CR/CRi rate of 44.0% in 16 ND FLT3^mut^ patients receiving VEN plus LDAC.^15^ In a retrospective study, it was found that the combination of VEN plus HMA treatment resulted in a CR/CRi rate of 60.0% in 50 FLT3^mut^ cases, including 17 cases of ND and 33 cases of R/R AML^13^. However, it was observed that over 30% of the FLT3^mut^ patients did not respond to VEN‐based regimens, which may be attributed to the clinical and genetic heterogeneity of patients. It is widely recognized that genetics play a significant role in predicting the response to AML treatments.^3^, ^9^, ^16^, ^17^ Unfortunately, the relationship between genetics and the efficacy of VEN‐based therapy in FLT3^mut^ patients remains unclear.

In this retrospective multicenter study, a total of 266 patients (127 ND AML and 139 R/R AML) who received VEN‐based regimens were enrolled to investigate the influence of FLT3 mutations on treatment efficacy. Additionally, we aimed to determine the clinical and genetic predictors of outcomes in FLT3^mut^ patients.

## 2 METHODS

### 2.1 Study design and patients

**FIGURE 1:**
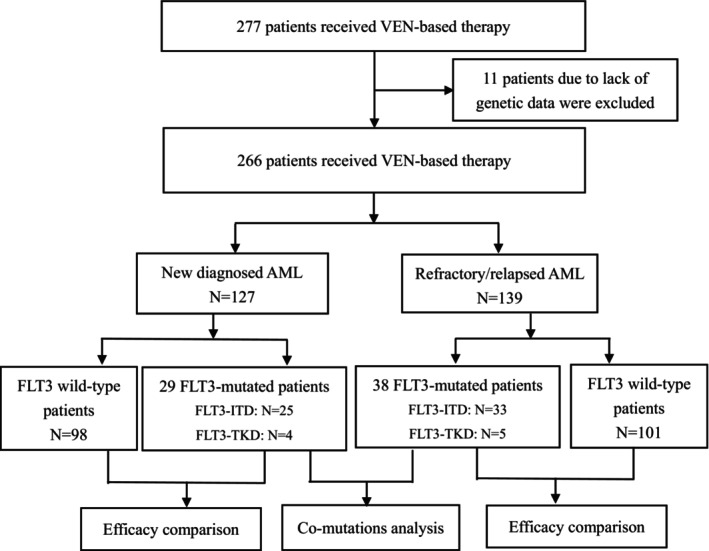
Flow chart. VEN, venetoclax; ND, newly diagnosed; R/R relapsed or refractory. Efficacy comparison was conducted between FLT3^mut^ and FLT3^wt^ patients (*N* = 266), and genetics analysis was only in FLT3^mut^ patients (*N* = 67).

In this retrospective study, we examined consecutive patients diagnosed as ND or R/R AML between July 2018 and April 2022 from 12 hospitals in southern China. These patients received at least one cycle of VEN‐based regimens. A total of 277 patients were identified as cases administrated with VEN‐based therapy. Eleven patients were excluded due to a lack of genetic data. Finally, 266 patients were enrolled, including 127 ND AML and 139 R/R AML. The treatment efficacy was compared between FLT3^mut^ and FLT3^wt^ patients, and co‐mutation analysis was conducted only in FLT3^mut^ patients. The detailed process of screening is depicted in Figure 1. AML was defined based on the World Health Organization's classification.^18^ Genetic risk was determined according to ELN risk stratification for 2022.^4^ R/R AML was defined the same as our previous study based on ELN risk stratification for 2017.^9^, ^19^ Data collection of genetics was conducted before the application of VEN therapy. As we previously reported,^9^ the standard metaphase karyotype and fluorescence in situ hybridization analysis were applied to evaluate cytogenetics. A 167‐gene panel (Table S1) and a 53‐gene PCR panel (Table S2) were used to detect mutations and fusion genes, respectively. Follow‐up data consisted of medical records of inpatients and outpatients and telephone records. The institutional review boards approved the ethics of study in accordance with the Declaration of Helsinki.

### 2.2 Assessment of response

Bone marrow (BM) assessment was done at 28 days after the initiation of VEN therapy. If BM was aplastic, the assessment was conducted again after hematologic recovery. After two cycles of VEN therapy or considered as clinically needed, BM assessments were also done. Disease responses were evaluated as per the 2022 European LeukemiaNet (ELN) criteria for AML and as previously described.^4^, ^9^ Disease responses include complete remission (CR), CR with incomplete hematological recovery (CRi), morphologic leukemia‐free state (MLFS), partial remission (PR), and non‐remission (NR). Detailed definitions of responses can be referred to in our previous study.^9^ Overall response (ORR) comprised CR, CRi, and MLFS, and CRc comprised CR and CRi. Non‐remission was defined as a failure to achieve PR. Once patients gained responses of CR/CRi, measurable residual disease (MRD) of BM aspirate samples was detected by multiparametric flow cytometry. The threshold of MRD was 0.01%. Besides, NPM1, RUNX1‐RUNX1T1, and CBFB‐MYH11 detected by PCR were also monitored as MRD.^20^

### 2.3 Statistical analysis

The continuous variables of the patients, summarized as range, median, or interquartile range, were analyzed using the Mann–Whitney *U* test. Categorical variables were summarized as frequencies or percentages and were analyzed using the chi‐squared test or Fisher's exact test. To identify the association of efficacy with clinical and genetic features, logistic regression was performed for treatment response, and Cox proportional hazards model was applied for survival. Overall survival (OS) was defined as the time from initiation of VEN to death, loss of follow‐up, and end of follow‐up. For survival analysis, Kaplan–Meier curves were applied to different subgroups and compared using log‐rank tests. Statistical significance was considered as *p* < 0.05 for a two‐tailed test. All data were analyzed using the EmpowerStats software and the statistical package R (http://www.r‐project.org). Graphs were plotted using GraphPad Prism 8.0.

## 3 RESULTS

### 3.1 Patient characteristics

**FIGURE 2:**
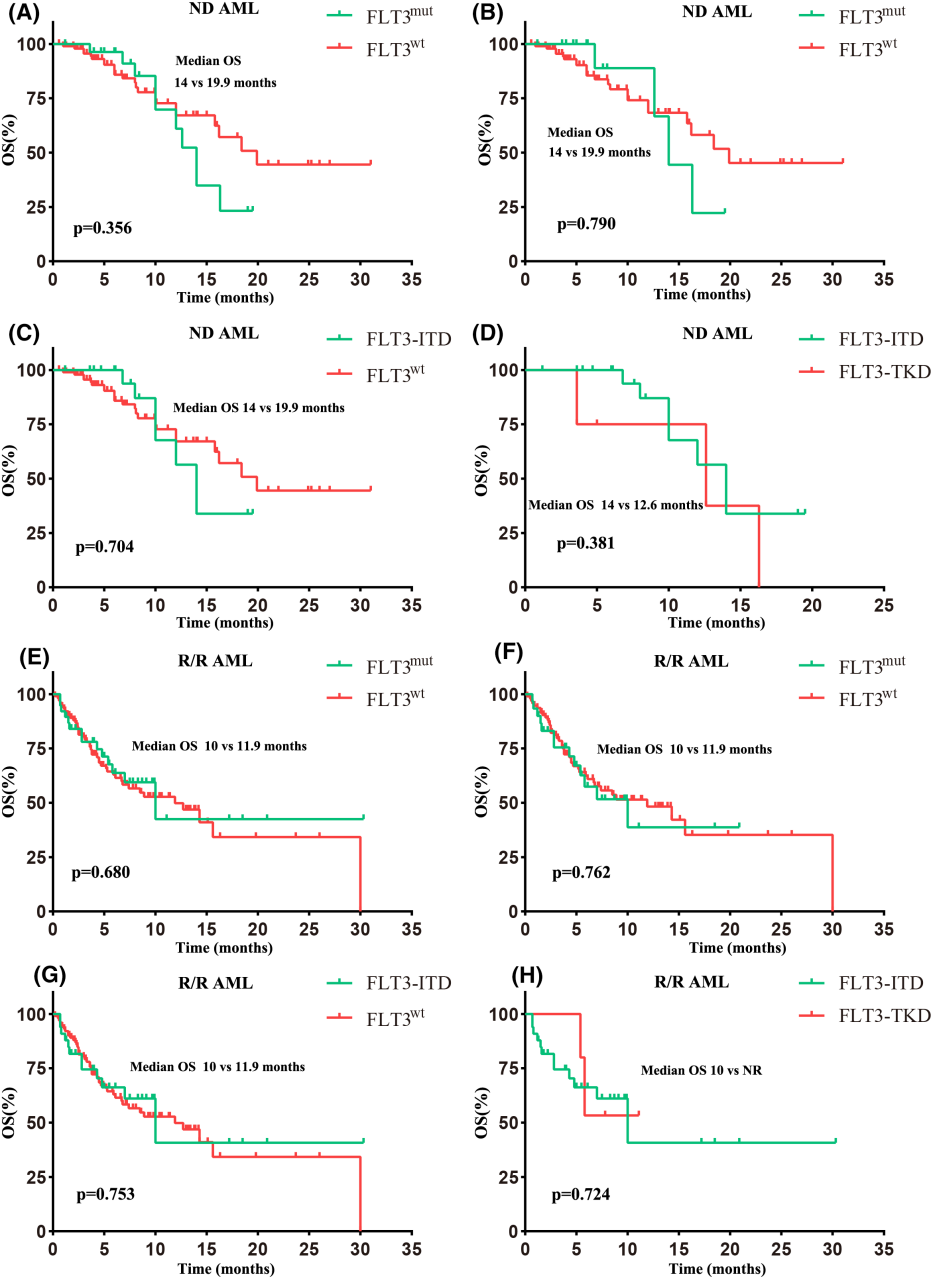
Survival. (A) OS based on FLT3 mutation in ND patients. (B) OS based on FLT3 mutation in patients without receiving sorafenib in ND patients. (C) OS based on FLT3‐ITD and FLT3^wt^ in ND patients. (D) OS based on FLT3‐ITD and ‐TKD in ND patients. (E) OS based on FLT3 mutation in R/R patients. (F) OS based on FLT3 mutation in patients without receiving sorafenib in R/R patients. (G) OS based on FLT3‐ITD and FLT3^wt^ in R/R patients. (H) OS based on FLT3‐ITD and ‐TKD in R/R patients. The start of follow‐up was from the first day of VEN therapy.

A total of 277 AML patients were assessed for eligibility, of whom, finally, 266 were enrolled, including 127 ND AML patients and 139 R/R AML patients. FLT3 mutations were detected (ND AML cohort vs. R/R AML cohort) in 29 versus 38 patients, and 98 versus 101 patients were FLT3 wild type, respectively. The study design and mutation landscape of patients are depicted in Figures 1 and 2, respectively. The key baseline and treatment characteristics are summarized in Table 1. For the ND AML cohort, a higher rate of WBC count and a lower rate of adverse cytogenetics were observed in the FLT3^mut^ group compared with the FLT3^wt^ group. For the R/R AML cohort, all variables were well‐balanced between the two groups.

**TABLE 1:** Patient baseline and treatment characteristics.

	ND AML			R/R AML		
	FLT3^mut^ (*N* = 29)	FLT3^wt^ (*N* = 98)	*p*‐Value	FLT3^mut^ (*N* = 38)	FLT3^wt^ (*N* = 101)	*p*‐Value
Gander, male/female, *N* (%)	12 (41.4)/17 (58.6)	59 (60.2)/39 (39.8)	0.073	18 (47.4)/20 (52.6)	56 (55.4)/45 (44.6)	0.395
Age (IQR)	64.0 (60.0–72.0)	65.0 (57.2–71.0)	0.874	55.5 (39.8–63.0)	52.0 (40.0–62.0)	0.746
ECOG score						
0–2	24 (82.8)	78 (79.6)	0.706	26 (68.4)	62 (61.4)	0.443
3–4	5 (17.2)	20 (20.4)		12 (31.6)	39 (38.6)	
AML type						
Denovo	25 (86.2)	69 (70.4)	0.088	14 (77.8)	41 (87.2)	0.344
Secondary	4 (13.8)	29 (29.6)		4 (22.2)	6 (12.8)	
White blood cell count, median (IQR; 10^9^/L)	33.5 (11.9–122.8)	6.6 (2.2–32.6)	0.002	14.1 (5.2–47.0)	9.3 (2.6–45.7)	0.189
BM blast, *N* (%)						
<30	4 (14.3)	26 (27.7)	0.08	1 (5.6)	14 (22.6)	0.217
30–50	6 (21.4)	30 (31.9)		4 (22.2)	15 (24.2)	
≥50	18 (64.3)	38 (40.4)		13 (72.2)	33 (53.2)	
Regimens, *N* (%)						
VEN + DAC	1 (3.4)	6 (6.1)	0.109	4 (10.5)	9 (8.9)	0.340
VEN + AZA	28 (96.6)	80 (81.6)		33 (86.8)	92 (91.1)	
VEN + AZA + HHT	0 (0.0)	12 (12.2)		1 (2.6)	0	
Prior intensive chemotherapy	0	0		25 (65.7)	60 (59.3)	0.342
Prior transplantation	0	0		8 (21.0)	25 (24.7)	0.269
Sorafenib for induction, *N* (%)	12 (41.4)	0		8 (21.1)	0	
Sorafenib posttransplantation, *N* (%)	2 (6.9)	0		7 (18.4)	0	
Cycles of VEN, median (IQR)	2.0 (1.0–4.0)	2.0 (1.0–3.0)	0.275	1.5 (1.0–3.0)	2.0 (1.0–3.0)	0.719
Bridge to allo‐HSCT, *N* (%)	5 (17.2)	16 (16.3)	0.907	9 (23.7)	25 (24.8)	0.896
Adverse cytogenetics, *N* (%)	2 (8.0%)	23 (28.0)	0.038	9 (23.7)	33 (32.7)	0.060

Abbreviations: AZA, azacitidine; BM, bone marrow; DAC, decitabine; ECOG, Eastern Cooperative Oncology Group; FLT3^mut^, FLT3 mutated; FLT3^wt^, FLT3 wild‐type; HHT, homoharringtonine;ND, new diagnosed; R/R, relapsed or refractory; VEN, venetoclax; WBC, white blood cells.

### 3.2 Treatment regimens and treatment responses

For induction therapy, 233 patients were treated with VEN + AZA, 20 were treated with VEN + DAC, and 13 were treated with VEN + AZA + HHT. Generally, these regimens consisted of VEN (100 mg on Day 1, 200 mg on Day 2, 400 mg on Days 3–28 for the VEN + AZA group; 100 mg on Day 1, 200 mg on Day 2, 400 mg on Days 3–14 for the VEN + AZA + HHT group), AZA (75 mg/m^2^/day on Days 1–7 subcutaneous) or DAC (20 mg/m^2^/day on Days 1–5 intravenous over 1 h), and HHT (1 mg/m^2^/day on Days 1–7 intravenous over 2 h). The dose of VEN was modified according to the tolerance to therapy and application of therapeutic or prophylactic anti‐infective agents.^2^ Twenty‐nine patients received sorafenib, including 20 during both induction and posttransplantation and 9 during posttransplantation only. Furthermore, 170 (64.0%) patients experienced sufficient time and dose of VEN during the first cycle. As a result of adverse events, 96 patients had dose interruptions and reductions in the first cycle. Fifty‐five patients received subsequent allo‐HSCT after induction therapy. Treatment details are presented in Table 1.

With a median of two cycles of VEN therapy, the outcomes are summarized in Tables 2 and 3 for the ND AML and R/R AML cohorts. For the ND AML cohort, CR/CRi rates were not significantly different not only between FLT3^mut^ and FLT3^wt^ groups (79.3% vs. 61.2%, *p* = 0.072) but also between FLT3‐ITD and FLT3^wt^ groups (80.0% vs. 61.2%, *p* = 0.079), respectively. MRD was detected in patients upon achieving CRc. Fifteen (65.2%) patients in the FLT3^mut^ group and 38 (63.3%) patients the FLT3^wt^ group were observed to be MRD‐negative (*p* = 0.842). Compared with FLT3^wt^ patients, a higher rate of relapse post‐CRc and shorter DOR were found in FLT3^mut^ patients (47.8% vs. 20.0%, *p* = 0.027; 8.6 vs. 15.6 months, *p* = 0.038), respectively. For the R/R AML cohort, compared with the FLT3^wt^ group, both FLT3^mut^ and FLT3‐ITD groups had lower CR/CRi rates (31.6% vs. 49.5%, *p* = 0.048; 27.3% vs. 49.5%, *p* = 0.026, respectively), and similar rates were observed for MRD‐negative cases (50.0% vs. 40.6%, *p* = 0.325; 44.4% vs. 40.6%, *p* = 0.654, respectively). Relapse rates post‐CRc of the FLT3‐ITD group were higher than that in the FLT3^wt^ group (41.7% vs. 31.7%, *p* = 0.045), while there was no significant difference between the FLT3^mut^ and FLT3^wt^ groups (40.6% vs. 31.7%, *p* = 0.075). Both FLT3^mut^ and FLT3‐ITD groups had shorter DOR than the FLT3^wt^ group (4.2 vs. 10.0 months, *p* = 0.041; 4.2 vs. 10.0 months, *p* = 0.048, respectively).

**TABLE 2:** Outcomes of ND AML patients.

Outcomes	FLT3^mut^ (*N* = 29)			FLT3^wt^ (*N* = 98)	*p*‐Value	*p*‐Value
	FLT3‐ITD (*N* = 25)	FLT3‐TKD (*N* = 4)	Total (*N* = 29)		FLT3^mut^ vs. FLT3^wt^	FLT3‐ITD vs. FLT3^wt^
Responses, *N* (%)						
ORR (CRc + MLFS)	21 (84.0)	4 (100.0)	25 (86.2)	65 (66.3)	0.038	0.085
CRc (CR + CRi)	20 (80.0)	3 (75.0)	23 (79.3)	60 (61.2)	0.072	0.079
CR	14 (56.0)	3 (75.0)	17 (58.6)	42 (42.9)	0.135	0.239
CRi	6 (24.0)	0	6 (20.7)	18 (18.4)	0.779	0.526
MLFS	1 (4.0)	1 (25.0)	2 (6.9)	5 (5.1)	0.710	0.819
MRD‐negative after CRc	12 (60.0)	3 (100.0)	15 (65.2)	38 (63.3)	0.842	0.754
Relapse						
Relapse rate post‐CRc, *N* (%)	9 (45.0)	2 (66.7)	11 (47.8)	12 (20.0)	0.027	0.036
DOR, median (range), months	8.6 (5‐NA)	8.8 (6.8‐NA)	8.6 (5.0‐NA)	15.6 (10.4‐NA)	0.025	0.038
Death						
Median follow‐up, months (range)	8 (6.1‐NA)		8 (6.1‐NA)	8 (6.8–11.2)	0.612	0.913
Died at the end of follow‐up, *N* (%)	7 (28.0)	3 (75.0)	10 (34.5)	23 (23.5)	0.213	0.721

Abbreviations: CI, confidence incidence; DOR, duration of remission; FLT3^mut^, FLT3 mutated; FLT3^wt^, FLT3 wild‐type; MRD, measurable residual disease; ND, new diagnosed; R/R, relapsed or refractory.

**TABLE 3:** Outcomes of R/R AML patients.

Outcomes	FLT3^mut^ (*N* = 38)			FLT3^wt^ (*N* = 101)	*p*‐Value	*p*‐Value
	FLT3‐ITD (*N* = 33)	FLT3‐TKD (*N* = 5)	Total (*N* = 38)		FLT3^mut^ vs. FLT3^wt^	FLT3‐ITD vs. FLT3^wt^
Responses, *N* (%)						
ORR (CRc + MLFS)	10 (30.3)	3 (60.0)	13 (34.2)	51 (50.5)	0.086	0.043
CRc (CR + CRi)	9 (27.3)	3 (60.0)	12 (31.6)	50 (49.5)	0.048	0.026
CR	4 (12.1)	1 (20.0)	5 (13.2)	27 (26.7)	0.090	0.084
CRi	5 (15.2)	2 (40.0)	7 (18.4)	23 (22.8)	0.578	0.350
MLFS	1 (3.0)	0	1 (2.6)	1 (1.0)	0.473	0.433
MRD‐negative after CRc	4 (44.4)	2 (66.6)	6 (50.0)	13 (40.6)	0.325	0.654
Relapse						
Relapse rate post‐CRc, *N* (%)	5 (41.7)	1 (33.3)	6 (40.0)	19 (31.7)	0.075	0.045
DOR, median (range), months	4.2 (2.8‐NA)	NA	4.2 (3.6‐NA)	10 (7.5‐NA)	0.041	0.048
Death						
Median follow‐up, months (range)	8.3 (5.5–18.5)	7.8 (7.8‐NA)	8.3 (6.1–17.2)	9.3 (7.1–11.4)	0.957	0.967
Died at the end of follow‐up, *N* (%)	13 (39.3)	2 (40.0)	15 (39.4)	44 (43.5)	0.754	0.786

Abbreviations: CI, confidence incidence; DOR, duration of remission; FLT3^mut^, FLT3 mutated; FLT3^wt^, FLT3 wild‐type; MRD, measurable residual disease; ND, new diagnosed; R/R, relapsed or refractory.

Subgroup analyses of CRc are presented in Figure S1. With regard to the ND AML cohort, in patients with IDH1/2 wild type and RUNX1 wild type, the FLT3^mut^ group achieved a superior response (CRc) than the FLT3^wt^ group (odds ratio [OR], 3.0; 95% confidence interval [CI], 1.0–9.4; *p* = 0.044; OR, 3.5; 95% CI: 1.1–11.0; *p* = 0.033; Figure S1A, respectively). In the R/R AML cohort, in patients with adverse ELN risk and DNMT3A mutation, the FLT3^mut^ group exhibited an inferior response (CRc) than the FLT3^wt^ group (OR, 0.3; 95% CI: 0.1–1.0; *p* = 0.043; OR, 0.2; 95% CI: 0.1–0.7; *p* = 0.016; Figure S1B, respectively). Consistent results were observed in patients with K/NRAS wild type and NPM1 wild type (OR, 0.4; 95% CI: 0.2–0.9; *p* = 0.026; OR, 0.2; 95% CI: 0.1–0.8; *p* = 0.016, respectively). The results are encapsulated in Tables S3 and S4, with the effect of sorafenib on treatment response excluded. Similar findings were observed in the condition of excluding sorafenib.

### 3.3 Survival

Of 266 patients, with a median follow‐up of 8.6 months (95% CI: 8.0–10), 92 (34.6%) patients died, including 30 relapses, 42 disease progressions, 10 transplantation‐related complications, and 10 other causes.

In the ND AML cohort, the median OS in FLT3^mut^ and FLT3^wt^ groups were close (14.0 vs. 19.9 months, *p* = 0.356, Figure 2A; 14.0 vs. 19.9 months, *p* = 0.790, Figure 2B) regardless of whether the patients who received sorafenib were excluded or not. Patients with FLT3‐ITD presented a comparable median OS with patients with FLT3^wt^ and FLT3‐TKD (14.0 vs. 19.9 months, *p* = 0.704, Figure 2C; 14.0 vs. 12.6 months, *p* = 0.381, Figure 2D). The results of the median OS of the R/R AML cohort were consistent with that of the ND AML cohort. The median OS rates were not significantly different between FLT3^mut^ and FLT3^wt^ groups (Figure 2E,F), and the median OS of FLT3‐ITD patients was comparable with FLT3^wt^ or FLT3‐TKD patients (Figure 2G,H).

Subgroup analyses of survival are depicted in Figure S2. For the ND AML cohort, no significant difference was detected between FLT3^mut^ and FLT3^wt^ in any subgroup (Figure S2A). For the R/R AML cohort, in patients with DNMT3A mutation, FLT3^mut^ patients displayed inferior survival than FLT3^wt^ patients (hazard ratio [HR], 2.7; 95% CI: 1.0–7.2; *p* = 0.048; Figure S2B) and had superior survival than FLT3^wt^ patients in those without DNMT3A mutation (HR, 0.4; 95% CI: 0.1–0.9; *p* = 0.038; Figure S2B).

### 3.4 The predictors of treatment responses in FLT3^mut^ patients

**FIGURE 3:**
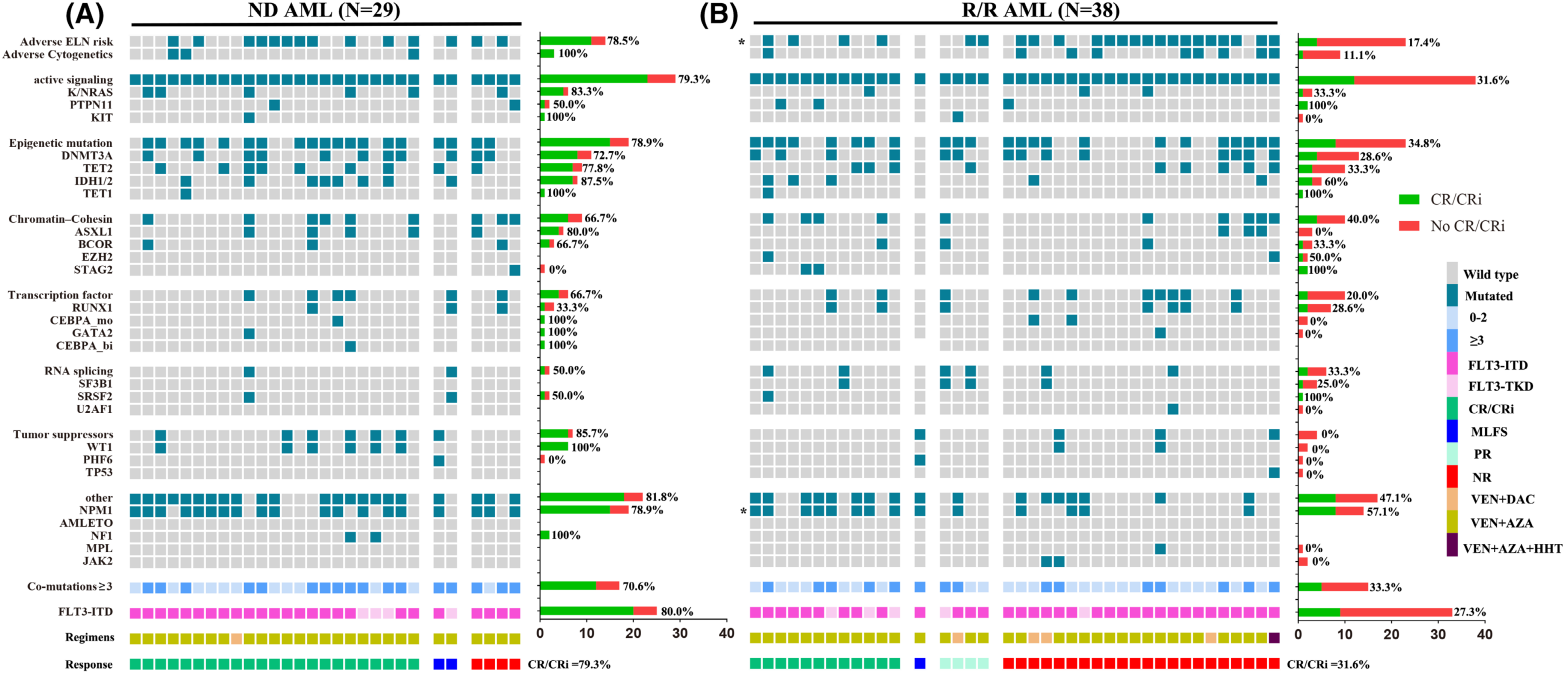
Genetic landscapes and treatment responses in FLT3^mut^ patients. Patients are grouped by the best response (CR/CRi, MLFS, PR, and NR), annotated with colored bars below the grid. The treatment regimens of each patient are indicated above response bar. The right side of the picture shows the number and composite complete remission (CRc) rate (green bar) of each genetic abnormality. Asterisks indicate genes with *p* < 0.05 for percent CRc. Active signaling: FLT3‐ITD, FLT3‐TKD, K/NRAS, PTPN11, and KIT; epigenetic mutations: TET2, DNMT3A, IDH1/2, and TET1; transcription factors: RUNX1, CEBPAmonallelic, GATA2, and CEBPAbiallelic; chromati‐cohesin: ASXL1, BCOR, EZH2, and STAG2; RNA‐splicing: SF3B1, SRSF2, and U2AF1; and tumor suppressors: TP53, WT1, and PHF6. (A) Genetic landscapes and treatment responses in ND AML patients. (B) Genetic landscapes and treatment responses in R/R AML patients.

The clinical characteristics of FLT3^mut^ patients are summarized in Table 1. The genetic landscapes of FLT3^mut^ patients are presented in Figure 3. Of the 67 patients (ND plus R/R), three exhibited 17p−, two had 5q−, seven presented complex or monosomal karyotype, and 60 harbored at least one co‐mutation. The most frequent co‐mutations (frequency ≥ 10%) included NPM1 (35.8%), DNMT3A (35.8%), TET2 (28.4%), IDH1/2 (19.4%), RUNX1 (14.9%), K/NRAS (13.4%), WT1 (11.9%), and ASXL1 (11.9%). The corresponding CR/CRi rates are summarized in Figure 3A,B. In the ND AML cohort, there were no clinical and genetic predictors of response for FLT3^mut^ patients (Figure 3A and Table S4). Notably, we observed a trend toward an inferior response to VEN therapy in patients with RUNX1 co‐mutation (CR/CRi rate: 33.0%; OR, 0.1; 95% CI: 0.0–1.3; *p* = 0.074; Figure 3A and Table S4). In the R/R AML cohort, adverse ELN risk was associated with an inferior response (CR/CRi rate = 17.4%; OR, 0.2; 95% CI: 0.1–0.8; *p* = 0.025; Figure 3B and Table S5). Furthermore, NPM1 co‐mutation was associated with a superior response (CR/CRi rate = 57.1%; OR, 6.7; 95% CI: 1.5–30.1; *p* = 0.014; Figure 3B and Table S5).

### 3.5 The predictors of survival in FLT3^mut^ patients

**FIGURE 4:**
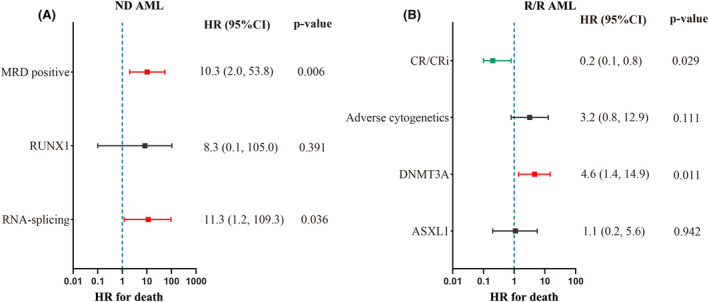
Multivariate analysis of predictors of survival. *Variables of *p* < 0.1 in univariate analysis (Tables S6 and S7) were included in this multivariate logistics model. (A) Predictors of survival in ND AML patients. (B) Predictors of survival in R/R AML patients.

**FIGURE 5:**
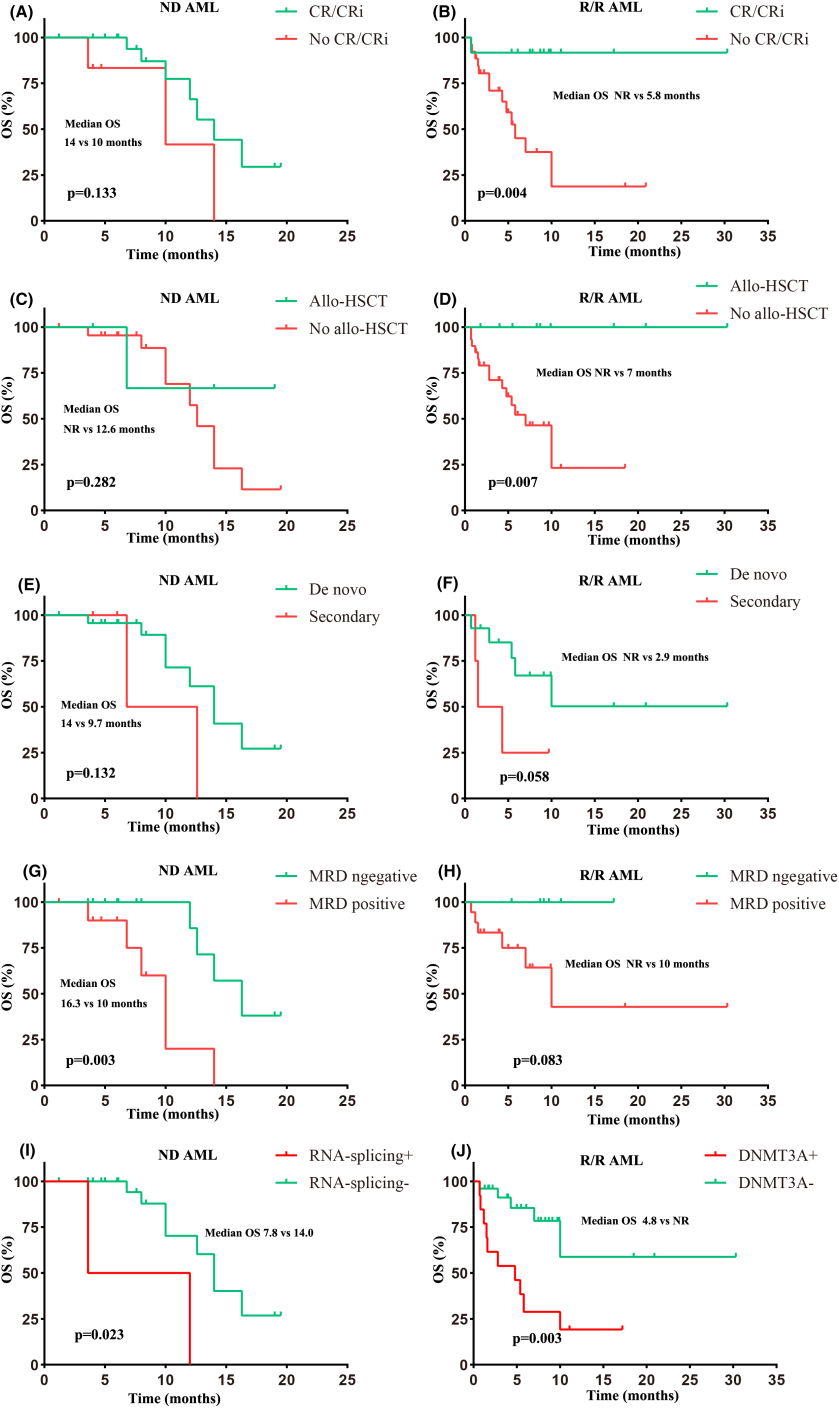
Survival of FLT3^mut^ patients. (A) OS based on CR/CRi in ND patients. (B) OS based on CR/CRi in R/R patients. (C) OS based on allo‐HSCT in ND patients. (D) OS based on allo‐HSCT in R/R patients. (E) OS based on AML type in ND patients. (F) OS based on AML type in R/R patients. (G) OS based on MRD in ND patients. (H) OS based on MRD in R/R patients. (I) OS based on RNA‐splicing in ND patients. (J) OS based on DNMT3A in R/R patients. The start of follow‐up was from the first day of VEN therapy.

In the ND AML cohort, univariate analysis indicated that MRD‐positive, RNA‐splicing, and SRSF2 mutations were risk factors for survival (Table S6). Multivariate analysis indicated that MRD‐positive and RNA‐splicing mutations were independent risk factors for survival (HR, 10.3; 95% CI: 2.0–53.8; *p* = 0.006; HR, 11.3; 95% CI: 1.2–109.3; *p* = 0.036; respectively; Figure 4A). The median OS of MRD‐positive and RNA‐splicing+ patients was shorter than that in MRD‐negative and RNA‐splicing− individuals (10.0 vs. 16.3 months, *p* = 0.003; 7.8 vs. 14.0 months, *p* = 0.023; Figure 5G,I). In the R/R AML cohort, univariate analysis indicated that CR/CRi was associated with improved survival, whereas adverse cytogenetics, DNMT3A, ASXL1, and TP53 mutations were associated with inferior survival (Table S7). Multivariate analysis indicated that CR/CRi predicted a lower risk of death (HR 0.2; 95% CI: 0.1–0.8; *p* = 0.029) and DNMT3A mutation predicted a higher risk of death (HR, 4.6; 95% CI: 1.4–14.9; *p* = 0.011; Figure 4B). The median OS was longer in patients achieving CR/CRi (NR vs. 5.8 months, *p* = 0.004; Figure 5B) and shorter in patients with DNMT3A mutation (4.8 vs. NR months, *p* = 0.003; Figure 5J). In addition, allo‐HSCT might improve the survival of R/R AML patients (NR vs. 7.0 months, *p* = 0.007; Figure 5D). AML type was not associated with survival in both ND and R/R AML patients (Figure 5E,F).

## 4 DISCUSSION

A comprehensive understanding of the influence of FLT3 mutations on the efficacy of VEN‐based therapy in patients with ND or R/R AML remains lacking. Furthermore, clinical and genetic predictors of the response and survival for FLT3^mut^ patients are undetermined. Our findings indicate that FLT3 mutations may affect the response to VEN‐based therapy in patients with R/R AML. Additionally, clinical and genetic characteristics have the potential to serve as prognostic indicators for the outcomes in AML patients with FLT3 mutations.

As a subset representing poor prognosis,^10^, ^21^, ^22^, ^23^ FLT3^mut^ patients have been highly concerned these years, especially in the era of targeted therapies. Previous studies have suggested that CRc rates of VEN‐based therapy for FLT3^mut^ patients vary greatly, ranging from 42.0% to 66.7%.^1^, ^2^, ^3^, ^13^, ^14^, ^15^, ^24^ These responses to VEN‐based therapy were associated with patients' characteristics, different combined regimens, and patients' genetic features. Regarding the disease setting of patients, Aldoss et al.^13^ suggested a CRc rate of 94.0% in ND AML patients and only 42.0% in R/R AML patients. Our results demonstrated that ND AML patients had a significantly higher CRc rate than R/R patients (79.3% vs. 31.6%), which was consistent with Aldoss et al.'s study and our previous study.^9^ With regard to combined regimens, in ND AML patients receiving VEN + HMA, as per the results of most studies, the CRc rates were from 55.5% to 72.0%^1^, ^2^, ^3^, ^14^, ^24^ in FTL3^mut^ patients. In ND AML patients treated with VEN + LDAC, Wei et al. showed a CRc rate of 44.0% in FLT3^mut^ patients. Our results were in general agreement with the former, with a CRc rate of 79.3%. Though these studies reported the CRc rates in ND or R/R AML patients with FLT3^mut^, only Konopleva et al.^14^ compared the CRc rates between FLT3^mut^ and FLT3^wt^ ND patients pooled from a randomized Phase 3 study. They found that FLT3^mut^ patients had comparable CRc rates with FLT3^wt^ patients (66.7% vs. 66.8%). Our results also suggested that the two groups of ND patients had similar CRc rates, consistent with Konopleva et al.'s findings. However, a new finding in our study is that FLT3^mut^ patients had higher CRc rates than FLT3^wt^ patients in the subgroups without IDH1/2 or RUNX1 mutations, which implied that these mutations may impact the treatment response of FLT3^mut^ patients. We found that FLT3^mut^ patients exhibited an inferior response compared with FLT3^wt^ patients in the R/R AML cohort, which indicated that FTL3 mutation had a negative impact on response to VEN‐based therapy in these patients. However, this impact was not observed in the aspect of survival. To our knowledge, this is the first real‐world study comparing the efficacy between FLT3^mut^ and FLT3^wt^ patients, including ND AML and R/R AML individuals. The impact of genetic heterogeneity on response has been documented in patients with ND or R/R AML.^3^, ^5^, ^6^, ^7^, ^9^, ^24^, ^25^ Nevertheless, this is still not well explored in the subset of FLT3^mut^ patients. In the total population, mutations in IDH1/2 were previously reported to be associated with a superior response to VEN‐based therapy in both ND and R/R AML patients. For ND AML patients, DiNardo et al. reported CRc rates of 75.4%^2^ and 90.9%,^3^ and Pollyea et al. reported CRc rates of 79.0%^26^ and 90.9%^24^ in patients with IDH1/2 mutations. For R/R AML patients, Stahl et al.^5^ found that patients with IDH1/2 had a CRc rate of 50.0%, higher than non‐IDH1/2 patients. In our previous study, we analyzed 150 patients with R/R AML and noted that IDH1/2 mutations were independent predictors of superior response.^9^ Regrettably, the association between IDH1/2 mutations and response to VEN‐based regimens was not well understood in the subset of FLT3^mut^ patients. Heretofore, it has only been reported by Konopleva et al.,^14^ who found that mutations in IDH1/2 were unfavorable factors of response to VEN regimens for FLT3^mut^ patients, with a CRc rate of 14% (1/7). Contrastingly, our results suggested that co‐mutations in IDH1/2 could not predict the treatment response of FLT3^mut^ patients in both ND AML and R/R AML cohorts. Therefore, further studies with a larger cohort will be needed to confirm and extend the conclusion of this study. NPM1 mutation was a predictor of favorable prognosis in AML.^27^, ^28^, ^29^ In total population, previous reports had suggested that patients with NPM1 mutation presented a superior response to VEN regimens in both ND and R/R AML.^3^, ^5^, ^9^ In the subset of FLT3^mut^ patients, Konopleva et al.^14^ showed that patients with co‐mutation of NPM1 seemed to have a relatively higher CRc rate than those with the wild type of NPM1, with CRc rates of 70.0% and 58.0, respectively. Aldoss et al. reported^13^ a CRc rate of 69.0% (9 out of 13) in NPM1^mut^ patients, but there was no statistically significant difference from NPM1^wt^ patients. Our results indicated a CRc of 78.9% in ND NPM1^mut^ patients, without significant difference from NPM1^wt^ patients. Thus, the effect of NPM1 mutation on FLT3^mut^ patients remains controversial in ND AML patients. Interestingly, a new finding in our study is that NPM1 mutation was a favorable predictor of response in R/R AML patients with FLT3^mut^. Another new finding in this study is that patients with RUNX1 co‐mutation tended to exhibit an inferior response in the ND AML cohort but did not show any statistical significance, with a CRc of 33.3%. Aldoss et al. reported a CRc of 50.0%, which was higher than ours. The impact of RUNX1 on treatment response in FLT3^mut^ patients should be considered in further studies with a larger cohort in the future. Our previous study showed that adverse ELN risk was associated with a worse response to VEN regimens for R/R AML. Our results demonstrated that it was also a predictor of response in R/R FLT3^mut^ patients. However, Aldoss et al. and Konopleva et al. did not analyze the impact of ELN risk on response to VEN therapy.^13^, ^14^

FLT3^mut^ AML has been the focus of intense research these years in view of the characteristics of high prevalence, resistance to chemotherapy, easy relapse, and worse survival.^21^, ^22^, ^30^, ^31^, ^32^ In the setting of VEN‐based regimens, the impact of FLT3^mut^ on survival has been reported in some studies. Data pooled from Phase 3 trials in ND patients treated with VEN + AZA revealed that the median OS rates were comparable between FLT3^mut^ and FLT3^wt^ patients (12.5 months vs. 14.7 months, respectively).^14^ A retrospective study also showed that FLT3^mut^ did not change the median OS of R/R AML patients receiving VEN + HMA or LDAC^5^ (median OS not reported). Aldoss et al.^13^ conducted a retrospective study in 50 patients (17 ND and 33 R/R) with FLT3^mut^ and found that the median OS was 11.3 months. In this study, the median OS of patients with FLT3^mut^ was similar to FLT3^wt^ patients in both ND AML and R/R AML cohorts, which was in line with previous studies.^13^, ^14^ These might suggest that VEN‐based therapy might overcome the poor effect of FLT3 mutation on survival. Co‐mutations of FLT3‐ITD and DNMT3A had been validated to be associated with worse OS before the emergence of VEN‐based therapy.^33^, ^34^ Our results first reported that DNMT3A mutation was an independent unfavorable factor of survival in FLT3^mut^ patients receiving VEN‐based therapy. This might imply that VEN‐based therapy might not overcome the effect of DNMAT3A mutation on the prognosis of FLT3^mut^ patients.

Our study has a few limitations that should be acknowledged. First, the retrospective nature and possible patient selection bias could not be avoided. Second, the small sample size may have led to bias. Third, the heterogeneity in treatments led to incompletely reliable conclusions. It should be noted that we still observed similar results when patients receiving sorafenib were excluded. To further validate these findings, a prospective and larger sample‐size study with longer follow‐up is needed in the future.

In conclusion, our findings indicate that FLT3 mutations exert a significant influence on the efficacy of VEN‐based therapy in patients with R/R AML. Moreover, the presence of IDH1/2 and NPM1 mutations may be indicative of a more favorable response in FLT3^mut^ patients, while an unfavorable ELN risk profile may be associated with a poorer treatment response. Additionally, the presence of adverse cytogenetics or a mutation in DNMT3A is predictive of worse survival in this patient population.

## AUTHOR CONTRIBUTIONS

**Guangyang Weng:** Data curation (equal); writing – original draft (equal); writing – review and editing (equal). **Jingya Huang:** Data curation (equal); writing – original draft (equal); writing – review and editing (equal). **Na An:** Data curation (equal); writing – original draft (equal); writing – review and editing (equal). **Yu Zhang:** Data curation (supporting); writing – review and editing (supporting). **Guopan Yu:** Data curation (supporting); writing – review and editing (supporting). **Zhiqiang Sun:** Data curation (supporting). **Dongjun Lin:** Data curation (supporting). **Lan Deng:** Data curation (supporting). **Xinquan Liang:** Data curation (supporting). **Jie Xiao:** Data curation (supporting). **Hongyu Zhang:** Data curation (supporting). **Ziwen Guo:** Data curation (supporting). **Xin He:** Data curation (supporting). **Hua Jin:** Data curation (supporting); writing – review and editing (supporting). **Qifa Liu:** Data curation (equal); supervision (equal); writing – review and editing (equal). **Xin Du:** Data curation (equal); supervision (equal); writing – review and editing (equal).

## CONFLICT OF INTEREST STATEMENT

The authors declare no competing interests.

## ETHICAL APPROVAL AND CONSENT TO PARTICIPATE

This retrospective study was approved by the ethics committee of Shenzhen Second People's Hospital and conducted in accordance with the Helsinki declaration. This retrospective study received a waiver for informed consent from the ethics committee of Shenzhen Second People's Hospital.

## Supporting information


Figure S1.

Figure S2.

Table S1.–S7.

## Data Availability

The datasets generated during and/or analyzed during the current study are available from the corresponding author on reasonable request.
