# Supplementary figures and images for: Clinical and genetic characteristics predict outcomes of acute myeloid leukemia patients with FLT3 mutations receiving venetoclax‐based therapy
Source: Cancer Med. 2024 Feb 9;13(2):e6885. doi: 10.1002/cam4.6885 (PMC10854448; doi:10.1002/cam4.6885)

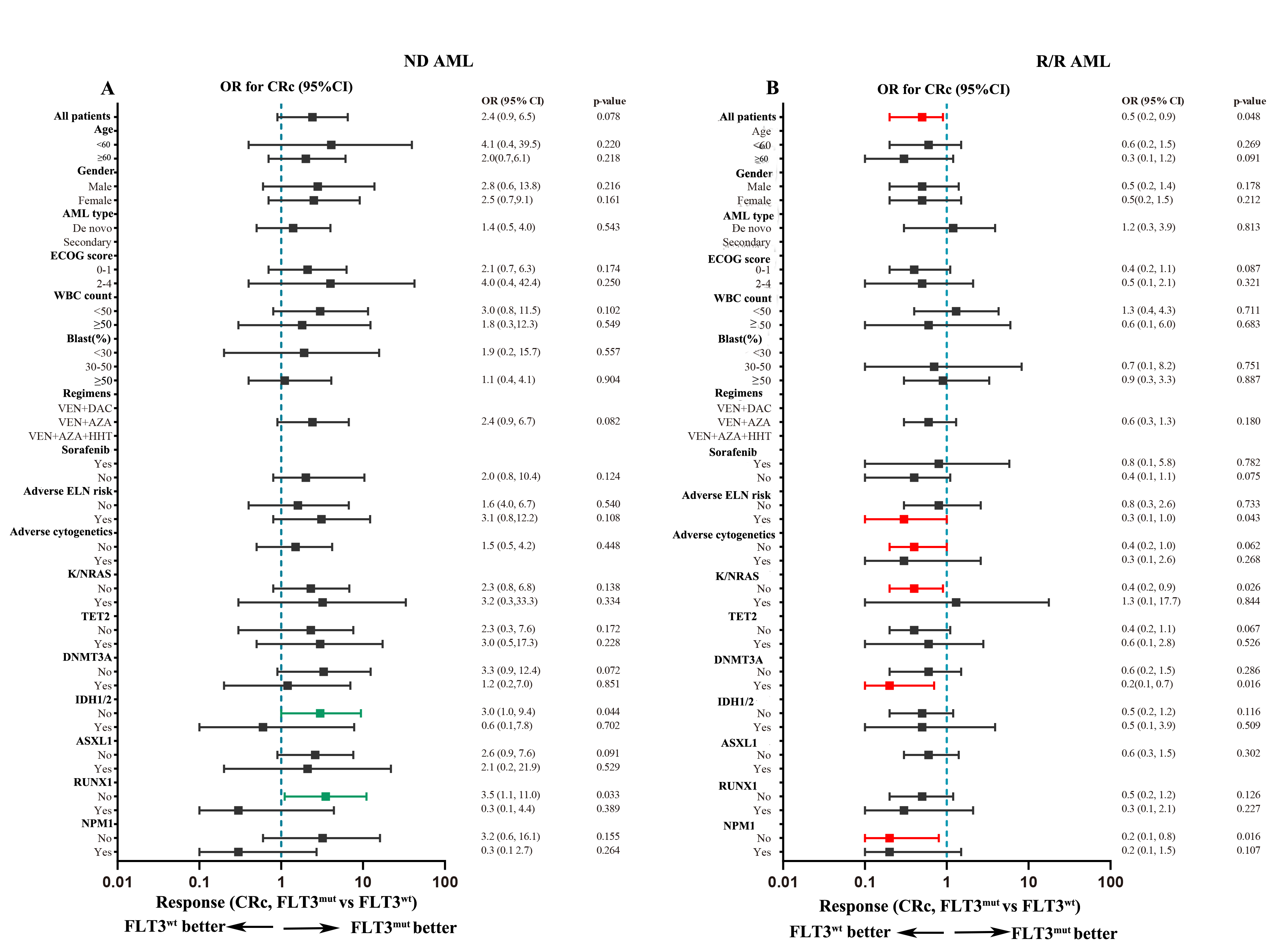

Supplement: Supplementary file 1 — Figure S1. [file CAM4-13-e6885-s002.tif]

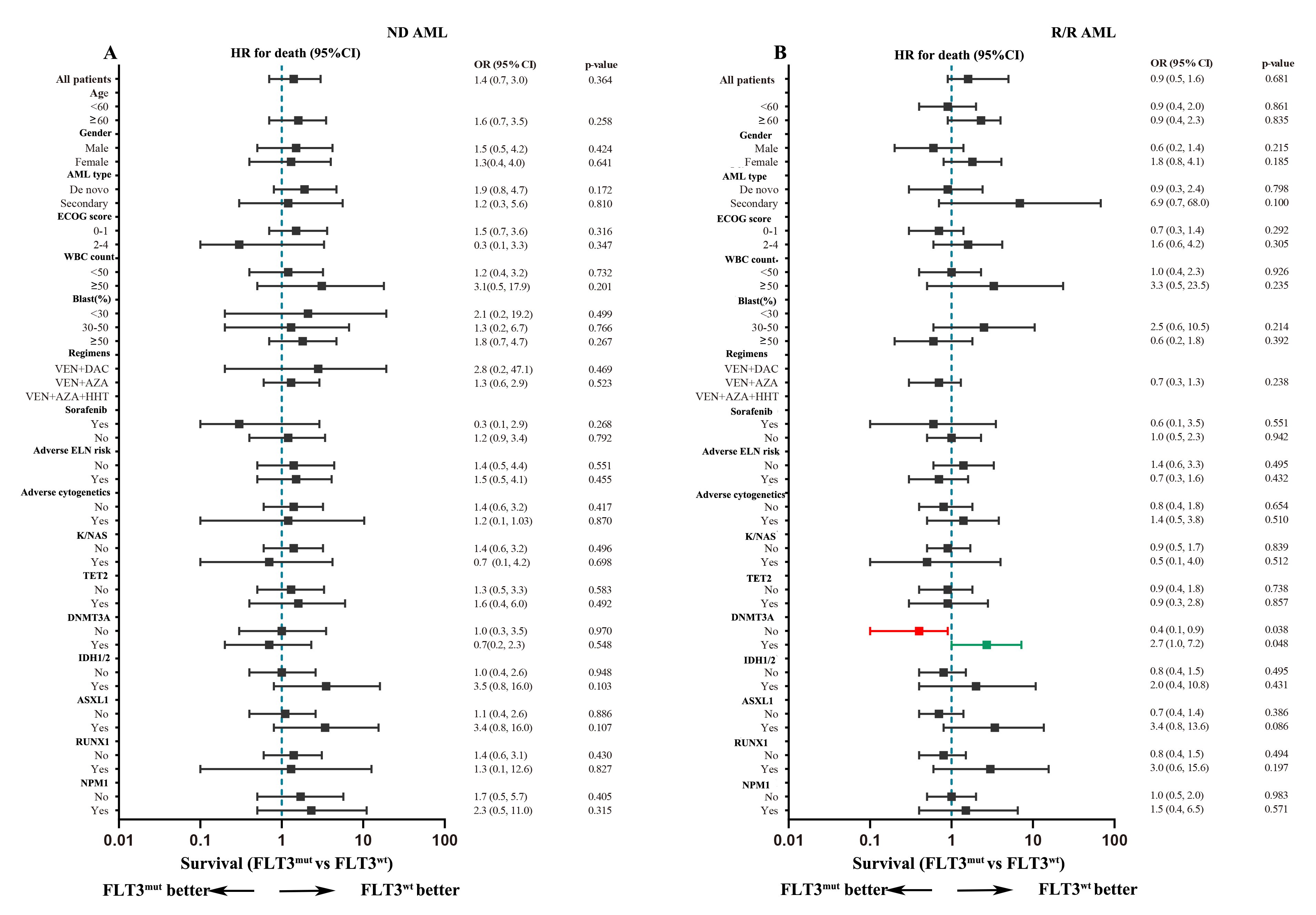

Supplement: Supplementary file 2 — Figure S2. [file CAM4-13-e6885-s001.tif]
