# Supplementary material for: Clinical and genetic characteristics predict outcomes of acute myeloid leukemia patients with FLT3 mutations receiving venetoclax‐based therapy
Source: Cancer Med. 2024 Feb 9;13(2):e6885. doi: 10.1002/cam4.6885 (PMC10854448; doi:10.1002/cam4.6885)
Supplement: Supplementary file 3 — Table S1.–S7. [file CAM4-13-e6885-s003.docx]

**Figure S1.** Subgroup analysis of response (CRc). (A) subgroup analysis in ND AML cohort. (B) subgroup analysis in R/R AML cohort. The dashed vertical line represents a hazard ratio of 1.0. Green or red bars represent statistical significance.

**Figure S2.** Subgroup analysis of survival. (A) subgroup analysis in ND AML cohort. (B) subgroup analysis in R/R AML cohort. The dashed vertical line represents a hazard ratio of 1.0. Green or red bars represent statistical significance.

**Table S1**. The 167-gene NGS panel.

| ABCA12 | CBL | CYLD | FAT4 | JAK3 | MYC | PRPF40B | SRSF2 | UBA2 |
| --- | --- | --- | --- | --- | --- | --- | --- | --- |
| ABL1 | CCND1 | DDB1 | FBXW7 | KDM2B | MYD88 | PTEN | SSPO | WHSC1 |
| ABL2 | CCND3 | DDX18 | FGFR3 | KDM5A | MYH11 | PTPN11 | STAG1 | WT1 |
| ACTR5 | CD101 | DDX3X | FLT3 | KDM6A | NF1 | PU.1 | STAG2 | XBP1 |
| AKT1 | CD123 | DIS3 | GATA1 | KIT | NFKB2 | RAD21 | STAT3 | XPO1 |
| ALK | CD79b | DNM2 | GATA2 | KRAS | NOTCH1 | RB1 | STAT5a | ZEB2 |
| ANK3 | CDC27 | DNMT1 | GATA3 | LMO1 | NOTCH2 | RELN | STAT5b | ZRSR2 |
| ARID1A | CDK4 | DNMT3A | GNAS | LMO2 | NPM1 | RHOA | SUZ12 |  |
| ARID2 | CDKN2A | EBF1 | HMGA2 | MAP2K1 | NRAS | ROS1 | SYK |  |
| ASXL1 | CDKN2B | ECT2L | HMGB1 | MDM2 | NT5C2 | RUNX1 | TAL1 |  |
| ATM | CEBPA | EED | HRAS | MEF2B | NUP98 | RUNX2 | TCF3 |  |
| ATRX | CHD1 | EGFR | ID2 | MLL | PAX5 | SAMHD1 | TERC |  |
| BAFF | CRBN | EP300 | IDH1 | MLL2 | PDGFRA | SETBP1 | TET1 |  |
| BCL2 | CREBBP | EPHA7 | IDH2 | MLL3 | PDGFRB | SETD2 | TET2 |  |
| BCL6 | CRLF2 | ERG1 | IKZF | MLL5 | PHF6 | SF1 | TIM-3 |  |
| BCOR | CSF1R | ETV6 | IL7R | MMD2 | PI3KCA | SF3A1 | TLX3 |  |
| BIRC3 | CSF3R | EVI1 | IRF4 | MN1 | PICALM | SF3B1 | TNFAIP3 |  |
| BRAF | CUX1 | EZH2 | IRF6 | MPL | PKM2 | SH2B3 | TP53 |  |
| CALR | CXCL12 | FAM46C | JAK1 | MTAP | PRDM1 | SOCS1 | TRAF3 |  |
| CARD11 | CXCR4 | FAT1 | JAK2 | MUM1 | PRMT5 | SOX4 | U2AF1 |  |

**Table S2.** The 53-gene PCR panel for fusion genes.

| TEL-ABL1 | MLL-AF6 | MLL-ENL | MLL-AF9 | MLL-AF4 |
| --- | --- | --- | --- | --- |
| MLL-ELL | MLL-AF1q | MLL-AF10 | MLL-AF7 | MLL-SEPT6 |
| MLL-AF17 | MLL-AF1p | RUNX1-RUNX1T1 | RUNX1-CBFA2T3 | E2A-PBX1 |
| E2A-HLF | PML-RARα | PLZF-RARα | NPM1-RARα | TLS-ERG |
| TEL-RUNX1 | TEL-PDGFRA | SIL-TAL | BCR-ABL1 | CBFβ-MYH11 |
| SET-NUP214 | DEK-NUP214 | TEL-JAK2 | ETV6-PDGFRA | NUP98-HOXA13 |
| NUP98-HOXC11 | NUP98-HOXD13 | NUP98-HOXA9 | NUP98-PMX1 | RUNX1-RPL22 |
| MLL-AFX1 | HOX11 | HOX11L2 | FIP1L1-PDGFRA | RUNX1-MDS1-EVI1 |
| DUP-MLL | MECOM | BCR-ABL190 | BCR-ABL210 | PML-RARα S |
| CALM-AF10 | PML-RARα V | PML-RARα L | STAT5B-RARα | NUMA1-RARα |
| FIPIL1-RARα | PRKARIA-RARα | NPM1-MLF |  |  |

**Table S3**. **Outcome of response in ND AML patients excluding those receiving sorafenib**

| **Outcomes** | FLT3mut(N=17) | | | FLT3wt (N=96) | **p-value** | **p-value** |
| --- | --- | --- | --- | --- | --- | --- |
|  | **FLT3-ITD (N=14)** | **FLT3-TKD (N=3)** | **Total (N=17)** |  | FLT3mut vs FLT3wt | FLT3-ITD vs FLT3wt |
| **Responses, N (%)** |  |  |  |  |  |  |
| ORR (CRc+MLFS) | 12 (85.7) | 3 (100.0) | 15 (88.2) | 65 (67.7) | 0.086 | 0.170 |
| CRc (CR+CRi) | 11 (78.6) | 3 (100.0) | 14 (82.4) | 60 (62.5) | 0.113 | 0.240 |
| CR | 6 (42.9) | 3 (100.0) | 9 (52.9) | 42 (43.8) | 0.483 | 0.950 |
| CRi | 5 (35.7) | 0 | 5 (29.4) | 18 (18.8) | 0.314 | 0.145 |
| MLFS | 1 (7.1) | 0 | 1 (5.9) | 5 (5.2) | 0.909 | 0.766 |

FLT3^mut^, FLT3 mutated; FLT3^wt^, FLT3 wild-type; ND, new diagnosed; R/R, relapsed or refractory; MRD, measurable residual disease; DOR, duration of remission;

**Table S4**. **Outcome of response in R/R AML patients excluding those receiving sorafenib**

| **Outcomes** | **FLT3^mut^(N=30)** | | | **FLT3^wt^ (N=94)** | **p-value** | **p-value** |
| --- | --- | --- | --- | --- | --- | --- |
|  | **FLT3-ITD (N=25)** | **FLT3-TKD (N=5)** | **Total (N=30)** |  | **FLT3^mut^ vs FLT3^wt^** | **FLT3-ITD vs FLT3^wt^** |
| **Responses, N (%)** |  |  |  |  |  |  |
| ORR (CRc+MLFS) | 6 (24.0) | 3 (60.0) | 9 (30.0) | 47 (50.0) | 0.055 | 0.020 |
| CRc (CR+CRi) | 5 (20.0) | 3 (60.0) | 8 (26.7) | 46 (48.9) | 0.032 | 0.009 |
| CR | 2 (8.0) | 1 (20.0) | 3 (10.0) | 24 (25.5) | 0.073 | 0.059 |
| CRi | 3 (12.0) | 2 (40.0) | 5 (16.7) | 22 (23.4) | 0.436 | 0.213 |
| MLFS | 1 (4.0) | 0 | 1 (3.3) | 1 (1.1) | 0.427 | 0.310 |

FLT3^mut^, FLT3 mutated; FLT3^wt^, FLT3 wild-type; ND, new diagnosed; R/R, relapsed or refractory; MRD, measurable residual disease; DOR, duration of remission;

**Table S4. Univariable analysis of predictors for response in ND AML patients with FLT3^mut^**

| **Variables** | **OR (95%CI)** | **p-value** |
| --- | --- | --- |
| Age |  |  |
| <60 | reference |  |
| ≥60 | 0.7 (0.1, 7.7) | 0.785 |
| Gender |  |  |
| Male | reference |  |
| Female | 0.6 (0.1, 4.3) | 0.655 |
| AML type |  |  |
| de novo | reference |  |
| secondary | inf. (0.0, Inf) | 0.996 |
| ECOG score |  |  |
| 0-1 | reference |  |
| 2-4 | 1.1 (0.1, 11.6) | 0.967 |
| WBC count |  |  |
| <50 | reference |  |
| ≥50 | 1.0 (0.1, 7.5) | 0.970 |
| Blast(%) |  |  |
| <30 | reference |  |
| 30-50 | inf. (0.0, Inf) | 0.994 |
| ≥50 | 3.5 (0.4, 33.3) | 0.276 |
| Regimens |  |  |
| VEN+DAC | reference |  |
| VEN+AZA | 0.0 (0.0, Inf) | 0.995 |
| Sorafenib |  |  |
| Yes | reference |  |
| No | 1.6 (0.3, 9.5) | 0.632 |
| Adverse ELN risk |  |  |
| No | reference |  |
| Yes | 0.9 (0.2, 5.5) | 0.924 |
| Adverse cytogenetics |  |  |
| No | reference |  |
| Yes | inf. (0.0, Inf) | 0.994 |
| K/NRAS |  |  |
| No | reference |  |
| Yes | 1.4 (0.1, 14.8) | 0.785 |
| Epigenetic mutations |  |  |
| No | reference |  |
| Yes | 0.9 (0.1, 6.3) | 0.947 |
| TET2 |  |  |
| No | reference |  |
| Yes | 0.9 (0.1, 5.9) | 0.891 |
| DNMT3A |  |  |
| No | reference |  |
| Yes | 0.5 (0.1, 3.3) | 0.497 |
| IDH1/2 |  |  |
| No | reference |  |
| Yes | 2.2 (0.2, 22.3) | 0.509 |
| Chromatin/cohesin |  |  |
| No | reference |  |
| Yes | 0.4 (0.1, 2.2) | 0.270 |
| ASXL1 |  |  |
| No | reference |  |
| Yes | 1.1 (0.1, 11.6) | 0.967 |
| Transcription factor |  |  |
| No | reference |  |
| Yes | 0.4 (0.1, 3.1) | 0.399 |
| RUNX1 |  |  |
| No | reference |  |
| Yes | 0.1 (0.0, 1.3) | 0.074 |
| RNA-splicing |  |  |
| No | reference |  |
| Yes | 0.2 (0.0, 4.3) | 0.323 |
| SRSF2 |  |  |
| No | reference |  |
| Yes | 0.2 (0.0, 4.3) | 0.323 |
| Tumor supperessors |  |  |
| No | reference |  |
| Yes | 1.8 (0.2, 18.3) | 0.634 |
| WT1 |  |  |
| No | reference |  |
| Yes | inf. (0.0, Inf) | 0.995 |
| PHF6 |  |  |
| No | reference |  |
| Yes | 0.0 (0.0, Inf) 0 | 0.994 |
| NPM1 |  |  |
| No | reference |  |
| Yes | 0.9 (0.1, 6.3) | 0.947 |

**Table S5. Univariable analysis of predictors for response in R/R AML patients with FLT3^mut^**

| **Variables** | OR for CRc | p-value |
| --- | --- | --- |
| Age |  |  |
| <60 | reference |  |
| ≥60 | 0.8 (0.2, 3.4) | 0.761 |
| Gender |  |  |
| Male | reference |  |
| Female | 0.9 (0.2, 3.4) | 0.825 |
| AML type |  |  |
| de novo | reference |  |
| secondary | 0.3 (0.0, 4.0) | 0.388 |
| ECOG score |  |  |
| 0-1 | reference |  |
| 2-4 | 0.6 (0.1, 2.9) | 0.555 |
| WBC count |  |  |
| <50 | reference |  |
| ≥50 | 1.2 (0.1, 11.0) | 0.893 |
| Blast(%) |  |  |
| <30 | reference |  |
| 30-50 | 0.0 (0.0, Inf) | 0.994 |
| ≥50 | 0.0 (0.0, Inf) | 0.994 |
| Regimens |  |  |
| VEN+DAC | reference |  |
| VEN+AZA | inf. (0.0, Inf) | 0.993 |
| VEN+AZA+HHT | 1.0 (0.0, Inf) | 1.000 |
| Sorafenib |  |  |
| Yes | reference |  |
| No | 0.3 (0.1, 1.7) | 0.183 |
| Adverse ELN risk |  |  |
| No | reference |  |
| Yes | 0.2 (0.1, 0.8) | **0.025** |
| Adverse cytogenetics |  |  |
| No | reference |  |
| Yes | 0.2 (0.0, 1.9) | 0.159 |
| K/NRAS |  |  |
| No | reference |  |
| Yes | 1.1 (0.1, 13.3) | 0.946 |
| Epigenetic mutations |  |  |
| No | reference |  |
| Yes | 1.5 (0.4, 6.1) | 0.600 |
| TET2 |  |  |
| No | reference |  |
| Yes | 0.9 (0.2, 4.3) | 0.900 |
| DNMT3A |  |  |
| No | reference |  |
| Yes | 0.9 (0.2, 4.0) | 0.938 |
| IDH1/2 |  |  |
| No | reference |  |
| Yes | 4.0 (0.6, 28.0) | 0.163 |
| Chromatin/cohesin |  |  |
| No | reference |  |
| Yes | 1.7 (0.4, 7.5) | 0.507 |
| ASXL1 |  |  |
| No | reference |  |
| Yes | 0.0 (0.0, Inf) | 0.994 |
| Transcription factor |  |  |
| No | reference |  |
| Yes | 0.5 (0.1, 2.5) | 0.366 |
| RUNX1 |  |  |
| No | reference |  |
| Yes | 0.8 (0.1, 5.1) | 0.850 |
| RNA-splicing |  |  |
| No | reference |  |
| Yes | 1.1 (0.2, 7.0) | 0.920 |
| U2AF1 |  |  |
| No | reference |  |
| Yes | 0.0 (0.0, Inf) | 0.995 |
| SRSF2 |  |  |
| No | reference |  |
| Yes | inf. (0.0, Inf) | 0.994 |
| SF3B1 |  |  |
| No | reference |  |
| Yes | 0.7 (0.1, 7.5) | 0.766 |
| Tumor supperessors |  |  |
| No | reference |  |
| Yes | 0.0 (0.0, Inf) | 0.993 |
| WT1 |  |  |
| No | reference |  |
| Yes | 0.0 (0.0, Inf) | 0.993 |
| TP53 |  |  |
| No | reference |  |
| Yes | 0.0 (0.0, Inf) | 0.995 |
| PHF6 |  |  |
| No | reference |  |
| Yes | 0.0 (0.0, Inf) | 0.995 |
| NPM1 |  |  |
| No | reference |  |
| Yes | 6.7 (1.5, 30.1) | **0.014** |

**Table S6**. **Univariate analysis of predictors for survival in ND AML patients with FLT3^mut^**

| **Variables** | **HR for death** | **p-value** |
| --- | --- | --- |
| Age |  |  |
| <60 | reference |  |
| ≥60 | inf. (0.0, Inf) | 0.999 |
| Gender |  |  |
| Male | reference |  |
| Female | 0.8 (0.2, 2.7) | 0.700 |
| AML type |  |  |
| de novo | reference |  |
| secondary | 3.3 (0.6, 17.2) | 0.159 |
| ECOG score |  |  |
| 0-1 | reference |  |
| 2-4 | 1.0 (0.2, 4.8) | 0.996 |
| WBC count |  |  |
| <50 | reference |  |
| ≥50 | 1.1 (0.3, 4.2) | 0.878 |
| Blast(%) |  |  |
| <30 | reference |  |
| 30-50 | 0.6 (0.0, 6.8) | 0.654 |
| ≥50 | 0.5 (0.1, 5.1) | 0.593 |
| REGIMENS |  |  |
| VEN+DAC | reference |  |
| VEN+AZA | 0.4 (0.0, 3.6) | 0.422 |
| Sorafenib |  |  |
| Yes | reference |  |
| No | 0.7 (0.2, 2.4) | 0.541 |
| MRD |  |  |
| Negative | reference |  |
| Positive | 6.7 (1.6, 27.9) | **0.009** |
| CR.CRI |  |  |
| No | reference |  |
| Yes | 0.4 (0.1, 1.5) | 0.164 |
| Bridge to allo-HSCT |  |  |
| No | reference |  |
| Yes | 0.3 (0.0, 2.8) | 0.320 |
| Adverse ELN risk |  |  |
| No | reference |  |
| Yes | 1.0 (0.3, 3.4) | 0.980 |
| Adverse cytogenetics |  |  |
| No | reference |  |
| Yes | 0.8 (0.1, 6.6) | 0.854 |
| K/NRAS |  |  |
| No | reference |  |
| Yes | 2.2 (0.5, 8.8) | 0.271 |
| Epigenetic mutations |  |  |
| No | reference |  |
| Yes | 1.0 (0.2, 5.1) | 0.971 |
| TET2 |  |  |
| No | reference |  |
| Yes | 2.1 (0.5, 7.9) | 0.285 |
| DNMT3A |  |  |
| No | reference |  |
| Yes | 1.6 (0.4, 6.3) | 0.530 |
| IDH1/2 |  |  |
| No | reference |  |
| Yes | 1.0 (0.3, 3.8) | 0.944 |
| Chromatin/cohesin |  |  |
| No | reference |  |
| Yes | 2.0 (0.5, 7.5) | 0.308 |
| ASXL1 |  |  |
| No | reference |  |
| Yes | 2.4 (0.6, 9.5) | 0.227 |
| Transcription factor |  |  |
| No | reference |  |
| Yes | 1.1 (0.2, 5.1) | 0.935 |
| RUNX1 |  |  |
| No | reference |  |
| Yes | 11.7 (0.7, 195.9) | **0.088** |
| RNA-splicing |  |  |
| No | reference |  |
| Yes | 5.6 (1.0, 31.3) | **0.048** |
| SRSF2 |  |  |
| No | reference |  |
| Yes |  | **0.048** |
| U2AF1 |  |  |
| No | NA |  |
| Yes | NA |  |
| SF3B1 |  |  |
| No | NA |  |
| Yes | NA |  |
| Tumor supperessors |  |  |
| No | reference |  |
| Yes | 0.0 (0.0, Inf) | 0.999 |
| PHF6 |  |  |
| No | reference |  |
| Yes | 0.0 (0.0, Inf) | 0.999 |
| WT1 |  |  |
| No | reference |  |
| Yes | 0.0 (0.0, Inf) | 0.999 |
| TP53 |  |  |
| No | NA |  |
| Yes | NA |  |
| NPM1 |  |  |
| No | reference |  |
| Yes | 0.5 (0.1, 2.0) | 0.307 |

**Table S7**. **Univariate analysis of predictors for survival in R/R AML patients with FLT3^mut^**

| Variables | HR for death | p-value |
| --- | --- | --- |
| Age |  |  |
| <60 | 1 |  |
| ≥60 | 1.3 (0.5, 3.8) | 0.571 |
| Gender |  |  |
| Male | 1 |  |
| Female | 1.4 (0.5, 3.9) | 0.541 |
| AML type |  |  |
| de novo | 1 |  |
| secondary | 3.9 (0.9, 18.0) | **0.078** |
| ECOG score |  |  |
| 0-1 | 1 |  |
| 2-4 | 2.2 (0.8, 6.3) | 0.135 |
| WBC count |  |  |
| <50 | 1 |  |
| ≥50 | 1.3 (0.3, 6.4) | 0.763 |
| Blast(%) |  |  |
| <30 | 1 |  |
| 30-50 | inf. (0.0, Inf) | 0.999 |
| ≥50 | inf. (0.0, Inf) | 0.999 |
| REGIMENS |  |  |
| VEN+DAC | 1 |  |
| VEN+AZA | 0.4 (0.1, 1.6) | 0.205 |
| VEN+AZA+HHT | 9.0 (0.7, 121.8) | 0.097 |
| Sorafenib |  |  |
| Yes | 1 |  |
| No | 2.1 (0.4, 10.3) | 0.342 |
| MRD |  |  |
| Negative | 1 |  |
| Positive | inf. (0.0, Inf) | 0.999 |
| CR.CRI |  |  |
| No | 1 |  |
| Yes | 0.1 (0.0, 0.7) | **0.022** |
| Bridge to allo-HSCT |  |  |
| No | 1 |  |
| Yes | 0.0 (0.0, Inf) | 0.998 |
| Adverse ELN risk |  |  |
| No | 1 |  |
| Yes | 0.9 (0.3, 2.4) | 0.776 |
| Adverse cytogenetics |  |  |
| No | 1 |  |
| Yes | 3.5 (1.1, 11.1) | **0.033** |
| K/NRAS |  |  |
| No | 1 |  |
| Yes | 0.8 (0.1, 6.4) | 0.856 |
| Epigenetic mutations |  |  |
| No | 1 |  |
| Yes | 4.5 (1.0, 20.0) | **0.048** |
| TET2 |  |  |
| No | 1 |  |
| Yes | 0.9 (0.3, 2.9) | 0.880 |
| DNMT3A |  |  |
| No | 1 |  |
| Yes | 4.5 (1.5, 13.3) | **0.006** |
| IDH1/2 |  |  |
| No | 1 |  |
| Yes | 1.2 (0.3, 5.4) | 0.827 |
| Chromatin/cohesin |  |  |
| No | 1 |  |
| Yes | 1.8 (0.6, 5.5) | 0.303 |
| ASXL1 |  |  |
| No | 1 |  |
| Yes | 6.5 (1.7, 25.2) | **0.007** |
| Transcription factor |  |  |
| No | 1 |  |
| Yes | 2.0 (0.7, 5.6) | 0.205 |
| RUNX1 |  |  |
| No | 1 |  |
| Yes | 1.1 (0.3, 3.9) | 0.896 |
| RNA-splicing |  |  |
| No | 1 |  |
| Yes | 0.8 (0.2, 3.1) | 0.800 |
| SRSF2 |  |  |
| No | 1 |  |
| Yes | 0.0 (0.0, Inf) | 0.998 |
| U2AF1 |  |  |
| No | 1 |  |
| Yes | 1.3 (0.2, 10.2) | 0.808 |
| SF3B1 |  |  |
| No | 1 |  |
| Yes | 0.9 (0.2, 4.3) | 0.937 |
| Tumor supperessors |  |  |
| No | 1 |  |
| Yes | 2.2 (0.5, 10.0) | 0.308 |
| PHF6 |  |  |
| No | 1 |  |
| Yes | 0.0 (0.0, Inf) | 0.998 |
| WT1 |  |  |
| No | 1 |  |
| Yes | 1.5 (0.2, 11.7) | 0.689 |
| TP53 |  |  |
| No | 1 |  |
| Yes | 18.2 (1.6, 200.3) | **0.018** |
| NPM1 |  |  |
| No | 1 |  |
| Yes | 0.7 (0.2, 1.9) | 0.437 |
